# A portrait of 2 nematodes in liver of their paratenic fish hosts, illustrating different immunological approaches

**DOI:** 10.1017/S003118202510053X

**Published:** 2025-08

**Authors:** Bahram Sayyaf Dezfuli, Emanuela Franchella, Flavio Pironi, Daniela Giannetto

**Affiliations:** 1 Department of Life Sciences and Biotechnology, University of Ferrara, Ferrara, Italy; 2 Department of Biology, Faculty of Science, Mugla Sitki Kocman University, Mugla, Turkey

**Keywords:** fish paratenic hosts, hepatic granuloma, innate immune cells, nematode larvae

## Abstract

Comparative histopathological and ultrastructural investigations were performed on the livers of 2 fish species, namely, flounder (*Platichthys flesus* (L.)) naturally infected with the nematode *Anisakis simplex* (*s.l.*) (Rudolphi, 1809) larvae (L3) and tuvira (*Gymnotus inaequilabiatus*) (Valenciennes, 1839) harbouring the nematode *Brevimulticaecum* sp. (L3) (Shikhobalova and Mozgovoi, 1952). The intensity of infection by *A. simplex* (*s.l.*) larvae (L3) in flounders ranged from 3 to 10 parasites per organ. The worms were encapsulated by the peritoneal visceral serosa on the external surface of the liver. Infected *P. flesus* livers showed hepatocyte cytoplasmic rarefaction and cell swelling. A few immune cell types, such as macrophages, limited numbers of mast cells (MCs), lymphocytes and some epithelioid cells, were observed within the granuloma. The intensity of infection by *Brevimulticaecum* sp. (L3) in *G. inaequilabiatus* ranged from 4 to over 340 larvae per organ, and the nematode larvae were encircled by round-to-oval granulomas. Each granuloma possessed 3 concentric layers of cells and tissue: an inner layer in close proximity to the *Brevimulticaecum* sp. (L3) cuticle, formed by densely packed layers of epithelioid cells showing several desmosomes between each other; a middle layer of numerous MCs entrapped in a thin fibroblast-connective mesh; and an outer layer of fibrous connective tissue with thin, elongated fibroblasts. High numbers of macrophages and macrophage aggregates were scattered within the granuloma. This is the first study to compare the cellular nature of granulomas and the immune responses in the livers of paratenic fish hosts of 2 nematode species.

## Graphical abstract

**Figure:**
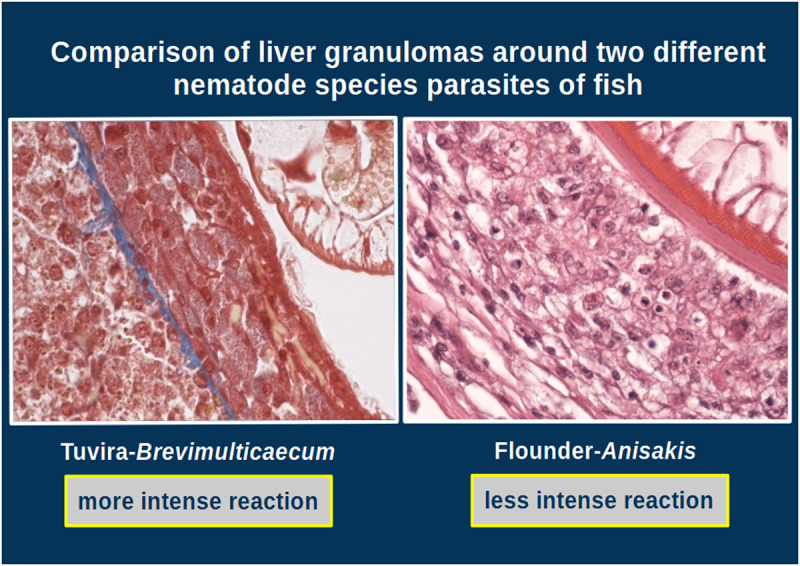

## Introduction

Intermediate and paratenic hosts are considered as helminth life cycle strategies (Parker et al. 2009). Paratenic hosts are those in which parasites do not grow or develop; they occur at some stage before the definitive host in the helminth life cycle (Bush et al. 2001) and are defined as incidental transport hosts or ‘ecological bridges’ that enhance transmission between hosts in a life cycle (Marcogliese 2001). Paratenic hosts exist in the life cycle of numerous helminth parasites of fishes. For example, nematodes of the *Anisakis* genus infect several species of marine organisms, with crustaceans as the first intermediate hosts, fishes and squids as intermediate and/or paratenic hosts (Køie M and Fagerholm. 1995), and cetaceans and one species of pinniped as definitive hosts (Shamsi et al. 2019; Mattiucci et al. 2020; Cipriani et al. 2024; Kumas et al. 2025).

Nematodes of the Anisakidae family are found mainly in fish-eating vertebrates (Køie et al. 1995; Cipriani et al. 2024). *Anisakis* larvae invade various tissues and organs of fish, such as the intestine, swimbladder, liver, gonads, somatic musculature, mesenteries and peritoneum (Moravec 1994; Sayyaf Dezfuli et al. 2021a). Anisakidae larvae occur encapsulated in/on the visceral organs of fish (Mehrdana and Buchmann 2017; Ying et al. 2022; Sayyaf Dezfuli et al. 2024), and many larvae frequently migrate from the visceral cavity into the fish flesh, posing a potential public health risk (Mattiucci et al. 2020; Shamsi and Barton 2023).

Species of the *Brevimulticaecum* genus have been identified in different regions of the world, and 6 species occur exclusively in South America (Santana et al. 2023). As with many nematode parasites of aquatic organisms, the *Brevimulticaecum* sp. life cycle involves intermediate, paratenic and definitive hosts. Some taxa of aquatic insects act as intermediate hosts (Isaac et al. 2004); amphibians, snakes and freshwater fish (e.g., *Gymnotus inaequilabiatus*) act as paratenic hosts (Vieira et al. 2010; Ventura et al. 2016); and crocodilians are definitive hosts (Santana et al. 2023).

Accounts dealing with the histopathology caused by tissue-penetrating Anisakidae nematodes in fishes are continuously increasing (Buchmann 2012; Buchmann and Mehrdana 2016;Sayyaf Dezfuli et al. 2017; Debenedetti et al. 2019; Molnár et al. 2019, 2021a; López-Verdejo et al. 2022). In fish liver infected with numerous nematode larvae, each parasite is encircled by a focal inflammatory granulomatous reaction (Noga 2010; Sayyaf Dezfuli et al. 2016; Molnár et al. 2019; Marnis et al. 2020). Granulomas on the surface of the internal organs of fishes are a common response to larval helminths (Buchmann and Mehrdana 2016; Sayyaf Dezfuli et al. 2021a; Behrens et al. 2023). Granulomas are focal chronic inflammatory lesions that appear as nodules in/on organs of the host (Molnár et al. 2019; Sayyaf Dezfuli et al. 2024). The following cell types might occur within the granuloma around a nematode larva: epithelioid cells (Molnár 1994; Sayyaf Dezfuli et al. 2017, 2024; Behrens et al. 2023), macrophages and macrophage aggregates (MAs) (Sayyaf Dezfuli et al. 2017; Stosik et al. 2019, 2021a), mast cells (MCs) (Sayyaf Dezfuli et al. 2016, 2021b), fibroblasts (Zuo et al. 2017; Molnár et al. 2019; Behrens et al. 2023), lymphocytes (Dezfuli et al. 2007; Behrens et al. 2023) and neutrophils (Ying et al. 2022).

Histopathology can be used to assess the health impacts of parasitism; however, ultrastructural observation of the liver is a superior tool for determining the health status of fish (Triebskorn et al. 2001). In this study, histopathological and ultrastructural analyses were performed on the livers of the flounder, *Platichthys flesus* (a brackish-water fish), and the tuvira, *G. inaequilabiatus* (a freshwater fish), which act as paratenic hosts for *Anisakis simplex* (*s.l.*) larvae (L3) and *Brevimulticaecum* sp. (L3), respectively. The structures of the granulomas around the larvae of the 2 nematode species were compared. This is the first study to compare the cellular nature of granulomas and the immune responses in the livers of paratenic fish hosts of 2 nematode species.

## Materials and methods

In January and March 2016, a subpopulation of 38 adult specimens of *G. inaequilabiatus* (mean total length ± standard deviation (SD): 32.36 ± 2.89 cm) from Porto Morrinho (21°41′56″S, 57°52′57″W), Brazil, was examined by researchers at the Federal University of Mato Grosso do Sul. The specimens were transported in oxygenated polyethylene bags to the laboratory facility of the Federal University where fishes were stocked for 2 h in an aquarium supplied with artificial aeration at constant temperature. Then fish were euthanized using 2-phenoxyethanol (2 ml/l) (Sigma-Aldrich, Hamburg, Germany) and then opened ventrally; all visceral organs were examined microscopically to identify helminth parasites. The liver of each fish was isolated from the rest of the alimentary canal and assessed under a stereomicroscope (Nikon SMZ800N, Tokyo, Japan) for the presence of encysted parasites on the organ surface or inside; the number of parasites was recorded. The liver of each parasitized specimen was fixed in 10% neutral buffered formalin for 24 h at 4°C, sliced into small pieces of 10 × 10 mm, rinsed several times with chilled 70% ethanol and sent to the University of Ferrara for embedding in paraffin wax. Multiple histological sections (5 µm thick) were obtained from each tissue block and stained using either haematoxylin and eosin (H&E) or Masson’s Trichrome staining. Some encysted larvae were isolated from a few heavily infected livers and fixed in 70% ethanol for parasite identification at the genus level.

Sixteen specimens of *P. flesus* (mean total length ± SD: 15.12 ± 4.70 cm) were collected from the River Forth (56°6’9″N 3°49’34″W), Stirling, Scotland. The fish were transported alive to the laboratory. The fish were given a lethal dose of 500 mg L^−1^ of the anesthetic MS222 (Sandoz, Basel, Switzerland), and their spinal cords were severed. During necropsy, the entire digestive tract was removed. The number and location of parasites were recorded, and some live larvae were isolated from the liver surface and fixed in 70% ethanol for species identification. Pieces of liver with attached nematodes, measuring up to 10 × 10 mm, were excised and fixed in 10% neutral buffered formalin for 24 h at 4°C. The samples were then transferred to 70% alcohol, dehydrated using an alcohol series and then processed routinely for paraffin embedding. Stained slides of the histological sections of the livers of both fish species were examined and photographed under an optical microscope (Nikon Eclipse 80i; Nikon, Tokyo, Japan).

For transmission electron microscopy (TEM), 7 × 7 mm pieces of infected livers of *G. inaequilabiatus* and *P. flesus* were fixed in chilled 2.5% glutaraldehyde in 0.1 M sodium cacodylate buffer for 3 h. The fixed tissues were post-fixed in 1% osmium tetroxide for 2 h, rinsed and stored in 0.1 M sodium cacodylate buffer containing 6% sucrose for 12 h. Thereafter, the tissue pieces were dehydrated using a graded acetone series and embedded in epoxy resin (Durcupan ACM, Fluka). Semi-thin sections (1.5 µm) were cut using a Reichert Om U2 ultramicrotome and stained with toluidine blue. Ultra-thin sections (90 nm) were stained with 4% uranyl acetate solution in 50% ethanol and Reynold’s lead citrate and then examined using a Talos L120C transmission electron microscope.

For both light microscopy and TEM, corresponding pieces of uninfected livers from both fish species were also processed so that a direct comparison with the infected material could be made.

## Results

### Light microscopy

**Figure 1.**
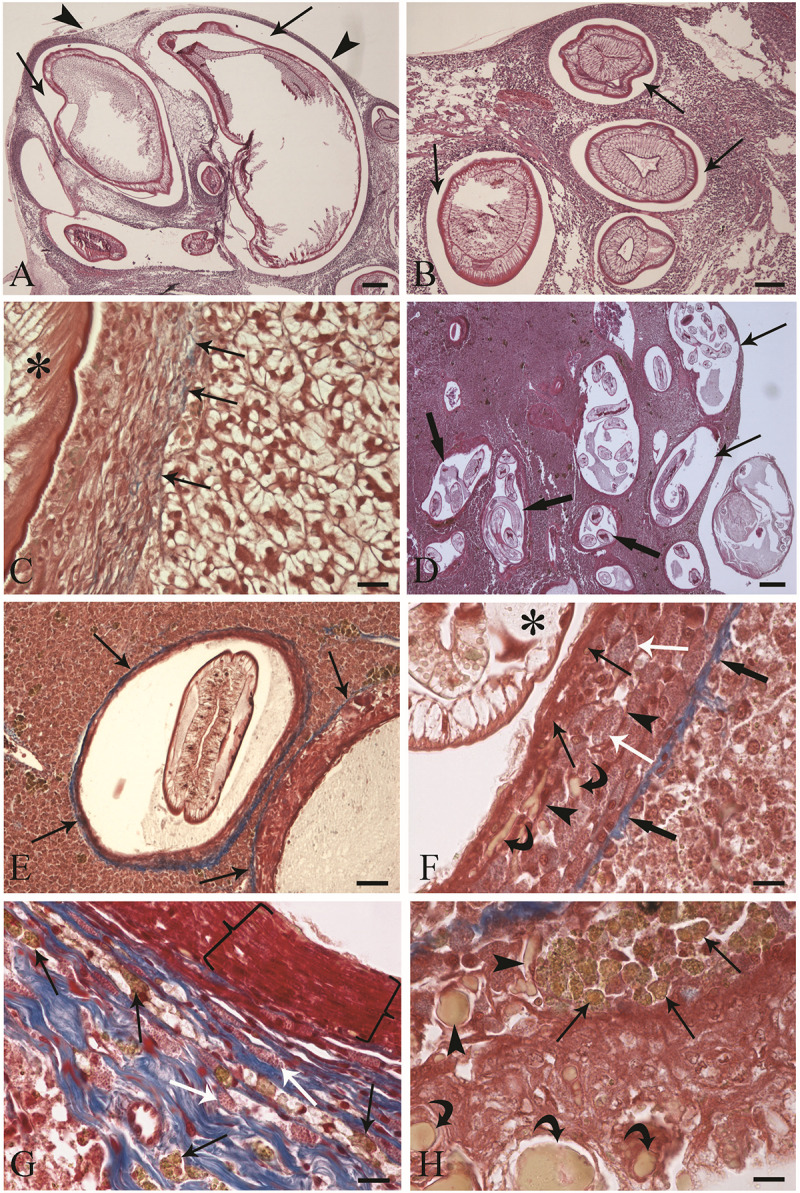
Histological sections of flounder (*Platichthys flesus*) liver infected with *Anisakis simplex* (*s.l.*) larvae (L3) and *Gymnotus inaequilabiatus* liver harbouring *Brevimulticaecum* sp. (L3). (A) *A. simplex* (*s.l.*) larvae (L3) in periphery of flounder liver encysted below the peritoneal visceral serosa (arrow heads). Translucent spaces (arrows) around larvae are visible; stain: haematoxylin–eosin (H&E); scale bar: 200 μm. (B) Some nematode larvae that penetrated deeply into the hepatic tissue. Translucent spaces (arrows) encircling parasite larvae are evident; stain: H&E; scale bar: 100 μm. (C) Micrograph showing *A. simplex* (*s.l.*) larvae (L3) cuticle (asterisk) near the granuloma wall and a low number of collagenous fibres (arrows) between hepatocytes and the outer layer of the granuloma; stain: Masson’s trichrome; scale bar: 25 μm. (D) Section of tuvira (*G. inaequilabiatus*) liver heavily infected with *Brevimulticaecum* sp. (L3). Few larvae (arrows) are in the organ’s periphery, and some (thick arrows) are encysted in deeper portions of the organ. There is no translucent space around each larva; stain: H&E; scale bar: 200 μm. (E) Encysted larvae encircled by granulomas, and a fibrous layer (arrows) separating the granulomas from hepatic tissue; stain: Masson’s trichrome; scale bar: 50 μm. (F) Micrograph showing details of layers of the granuloma around *Brevimulticaecum* sp. (L3). The inner layer of epithelioid cells (arrows) is in close proximity to the larva (asterisk). The middle layer comprises numerous mast cells (MCs; white arrows) surrounded by a thin fibroblast-connective mesh (arrow heads). Lipid droplets within the granuloma are visible (white arrow heads), and there is a fibrous layer (curved arrows) at the outer part of the granuloma; stain: Masson’s trichrome; scale bar: 50 μm. (G) Micrograph showing the inner part of the granuloma formed by several layers of epithelioid cells (brackets). Macrophages (arrows) scattered among abundant collagenous fibres are evident in the middle layer, along with MCs (white arrows); stain: Masson’s trichrome; scale bar: 10 μm. (H) A high number of macrophage aggregates (arrows) and lipid droplets (arrow heads) present in the outer layer of the granuloma, with small and big lipid droplets clustering in the liver parenchyma (curved arrows); stain: Masson’s trichrome; scale bar: 10 μm.

The livers of 8 out of 16 (50%) flounders harboured nematode larvae. Some larvae were removed from the liver surface, fixed in 70% ethanol and compared with regard to the morphological features of the *Anisakis* genus described by Moravec (1994); thus, the isolated worms were identified as *A. simplex* (*s.l.*) larvae (L3). The intensity of infection ranged from 3 to 10 worms per organ (5.5 ± 2.77, mean ± SD). All larvae on the liver surface were surrounded by granulomatous reactive tissue in the peritoneal visceral serosa (Figure 1A and 1B), and 8 larvae had penetrated the liver parenchyma and damaged the organ. At the site occupied by each larva, the hepatic tissue was replaced by nematodes (Figure 1A and 1B). A translucent space between the larval body and liver tissue was observed around the vast majority of the worms (Figure 1A and 1B); very rarely, *A. simplex* (*s.l.*) larvae (L3) were in close proximity to hepatocytes. A few collagenous fibres were observed between the granulomas and hepatic tissue (Figure 1C). Two livers infected with *P. flesus* exhibited fibrotic scarring. Reactive cellular elements within the granuloma around the *A. simplex* (*s.l.*) larvae (L3) were identified using TEM (refer to the subsection ‘Electron microscopy’).

The livers of 35 out of 38 (95%) examined *G. inaequilabiatus* specimens were infected with *Brevimulticaecum* sp. (L3) larvae; the intensity of infection varied from 4 to over 340 worms per organ (55.31 ± 73.94, mean ± SD). Nematodes of tuvira were identified as third-stage larvae of *Brevimulticaecum* sp. based on key features provided in (Moravec and Kaiser 1994).

The larvae were encysted below the visceral serosa and in the deeper part of the liver (Figure 1D) and were surrounded by granulomatous reactive tissue (Figure 1D and 1E). In heavily infected livers, parasitic larvae replaced most of the hepatic tissue (Figure 1D). Owing to the focal host tissue response to nematodes, each larva of *Brevimulticaecum* sp. (L3) was encircled by a round-to-oval-shaped granuloma (Figure 1D and 1E). Three concentric layers constituted the granuloma: an inner part adjacent to the nematode cuticle formed by a variable number of layers of epithelioid cells (Figure 1F and 1G), a middle layer of MCs entrapped in a thin fibroblast-connective mesh (Figure 1F) and an outer layer of fibrous connective tissue with elongated fibroblasts and collagenous fibres (Figure 1G). MCs exhibiting intense degranulation were frequently observed in the middle layer (refer to the subsection ‘Electron microscopy’). Macrophages were scattered in the liver parenchyma, and several were dispersed in the middle layer of the granuloma (Figure 1G); the presence of MAs was more remarkable adjacent to the outer layer (Figure 1H). Lipid droplets were present within the granuloma (Figure 1G) and liver parenchyma (Figure 1H). A fibrous layer of variable thickness was interposed between the outer layer of the granuloma and the surrounding hepatic tissue (Figure 1E and 1F). One to two fibrotic scars were observed in some heavily infected livers.

### Electron microscopy

**Figure 2.**
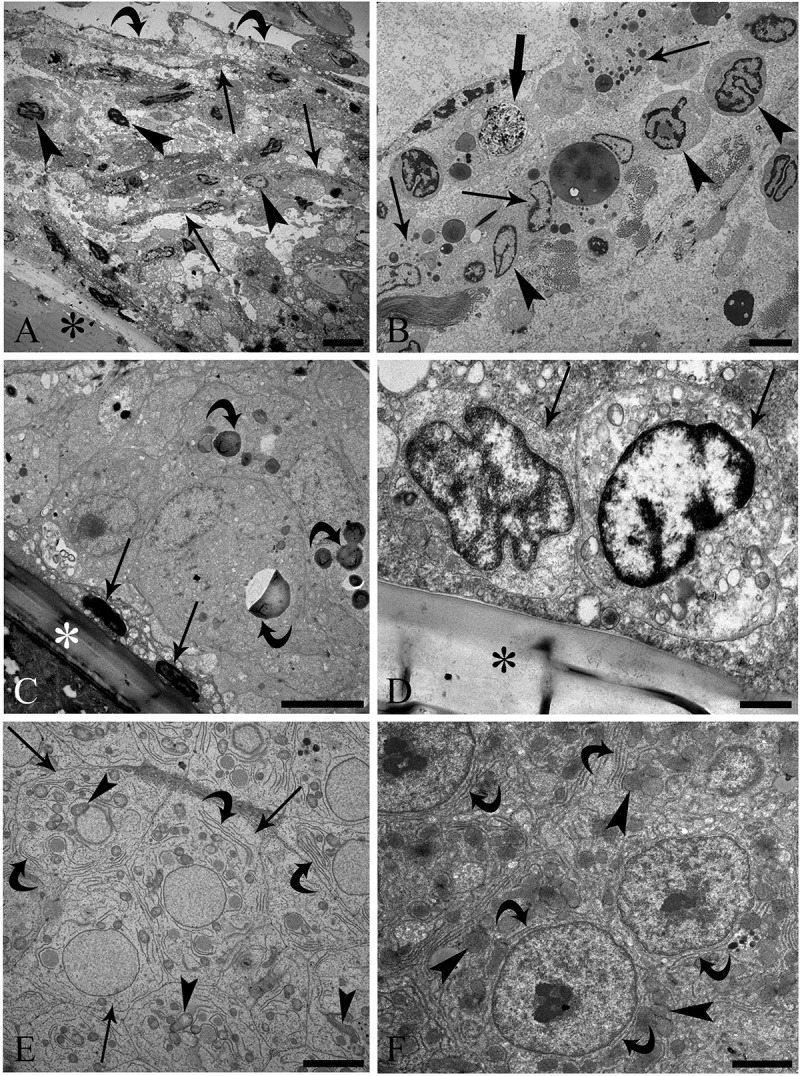
Transmission electron micrograph of flounder (*Platichthys flesus*) liver. (A) Interface region between *P. flesus* liver and *A. simplex* (*s.l.*) larva (L3). Nuclei of different types of immune cells (arrow heads) were scattered in a fibro-connective mesh (arrows) within the granuloma around the nematode larva (asterisk). The outer layer (curved arrows) with scarce collagen is visible; scale bar: 5 μm. (B) Aspect of the middle layer: a few mast cells (arrows), lymphocytes (arrow heads) and a macrophage (thick arrow) are evident; scale bar = 3.3 μm. (C) Inner layer: dark nuclei (arrows) of necrotic epithelioid cells near very big macrophages are in close proximity to the parasite cuticle (asterisk), with electron-dense vesicles inside the macrophages; scale bar: 5 μm. (D) Two lymphocytes (arrows) near nematode cuticle (asterisk); scale bar: 1 μm. (E) Micrograph of infected flounder liver: the hexagonal shape of hepatocytes (arrows) and rarefaction of their cytoplasm are clear, and dilatation of the mitochondria (arrow heads) and dispersion of rough endoplasmic reticulum (RER) cisternae (curved arrows) in the cytoplasm are evident; scale bar: 5 μm. (F) Image of uninfected liver: round hepatocytes, cytoplasm without rarefaction, well-developed RER (curved arrows) near nuclei and several mitochondria (arrow heads) are visible; scale bar: 2 μm.

The nuclei of different types of immune cells were scattered in the fibro-connective mesh within the majority of granulomas in infected *P. flesus* livers (Figure 2A). A corona of fibro-connective tissue with a scarce collagen component extended from the outer layers of the granulomas towards the larvae (Figure 2A). Very few MCs and several lymphocytes and macrophages were visible in the middle and inner layers (Figure 2B). The inner layer had numerous large macrophages in poor condition and containing undefinable electron-dense material (Figure 2C), whereas necrotic epithelioid cells (Figure 2C) and lymphocytes (Figure 2D) were observed very close to the nematode cuticle. In the parasitized livers, the hepatocytes were hexagonal-like in shape and swollen, and their cytoplasm lacked regular compartmentalization of organelles (Figure 2E). Nonetheless, the main alterations were mild rarefaction of the hepatocyte cytoplasm, mitochondrial dilatation and dispersion of rough endoplasmic reticulum (RER) cisternae in the cytoplasm, often far from the nucleus (Figure 2E). Figure 2F shows the normal aspects of an uninfected flounder liver, in which the hepatocytes are round in shape, with well-developed RER in close proximity to the nucleus, some normal mitochondria scattered in the cytoplasm and no cytoplasmic rarefaction.

**Figure 3.**
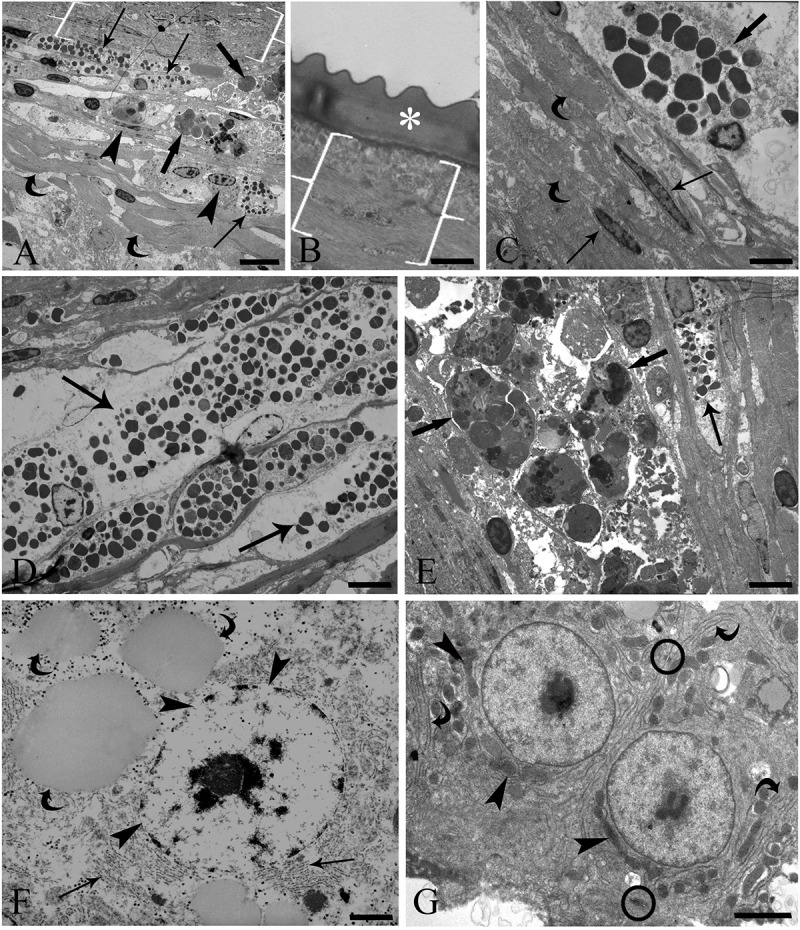
Transmission electron micrograph of *Gymnotus inaequilabiatus* liver. (A) The partition of the granuloma layers around *Brevimulticaecum* sp. (L3) is appreciable: the inner layer consists of epithelioid cells (brackets), the middle layer of mast cells (MCs; arrows), fibroblasts (arrow heads) and macrophage aggregates (MAs; thick arrows), and the outer layer of abundant connective fibres (curved arrows); scale bar: 5 μm. (B) High magnification of strict contact between nematode cuticle (asterisk) and inner layer of the granuloma (brackets); scale bar: 0.7 μm. (C) Image of confine region between inner and middle layers: 2 epithelioid cells (arrows) encircled by abundant collagenous fibres (curved arrows) and an MC (thick arrow) in the middle layer are visible; scale bar: 0.7 μm. (D) The presence of numerous MCs increased the thickness of the middle layer; some of these cells were degranulated (arrows); scale bar: 3.3 μm. (E) Micrograph of mainly middle layer, showing the presence of very big MAs (thick arrows). The MAs contain inclusions of differing electron densities with undefinable nature, along with an MC (arrow); scale bar: 3 μm. (F) Infected liver of tuvira with a hepatocyte having no evident plasmalemma. Rarefaction of the cytoplasm, interruption of the nucleoplasm (arrow heads), fragments of rough endoplasmic reticulum (RER; arrows) and lipid droplets (curved arrows) are evident; there are no mitochondria near the nucleus; scale bar: 1.1 μm. (G) Uninfected liver of tuvira showing 2 adjacent hepatocytes with desmosomes (circles) and well-developed RER (curved arrows). Numerous mitochondria (arrow heads) are close to the nuclei, and the lack of cytoplasmic rarefaction is appreciable; scale bar: 2 μm.

Ultrastructural observations of the cell types in granulomas in infected *G. inaequilabiatus* livers have been reported by Sayyaf Dezfuli et al. (2016); herein, only aspects that were not dealt with in previous records of the current authors are presented. The granulomas comprised 3 layers (Figure 3A); the inner region was often in strict contact with the cuticle of the nematode (Figure 3B) and was formed by some layers of cells, which often appeared darker than the elements of the other 2 layers (Figure 3B and 3C). These cells were elongated transformed macrophages, known as epithelioid cells, and were encircled by abundant collagenous fibres (Figure 3C). Numerous desmosomes were observed between the epithelioid cells (not shown). Figure 3C shows the demarcation line between the middle and inner layers; an MC is evident in the middle layer. The presence of numerous MCs, with several being degranulated, increased the thickness of the middle layer (Figure 3D). Very large MAs were mainly present in the middle layer (Figure 3E). The MAs consisted of groups of large, round-to-oval cells; their cytoplasm contained inclusions of differing electron densities of an uncertain nature (Figure 3E).

Near the granulomas of *G. inaequilabiatus* livers, hepatocytes appeared as large polyhedral cells with no evident plasmalemma but possessing large euchromatic nuclei. Nucleoplasm interruption, cytoplasmic rarefaction and dispersion of RER cisternae were observed (Figure 3F). Very few mitochondria were dilated, and cristae were absent (not shown). Lipid droplets were frequently observed (steatosis) in the hepatocytes of infected livers (Figure 3F). The histological and ultrastructural patterns of tuvira livers harbouring nematode larvae were suggestive of mild hepatocyte hydropic degeneration (Figure 3F). Figure 3H shows an uninfected liver of *G. inaequilabiatus* in which hepatocyte cytoplasmic rarefaction was totally absent, RER was well developed and several mitochondria with normal aspects were present near the nucleus; moreover, desmosomes were common between 2 hepatocytes.

## Discussion

Successful infection by helminths is mainly attributed to their capacity to evade and/or manipulate host immune systems (Secombes and Chappell 1996; Franke et al. 2014). Nematode parasites of the fish liver harm the organ in different ways. They can induce mechanical injury (Santoro et al. 2013; Molnár et al. 2019; Sayyaf Dezfuli et al. 2021a; Behrens et al. 2023) and affect the physiology of hepatic tissue and its capacity to store energy reserves (Podolska et al. 2024). Body condition factors and the hepatosomatic index significantly decrease with an increase in infection density (Podolska et al. 2024). The phylum Nematoda is the fifth most described among metazoan phyla. Approximately 30 000 species have been validated; nevertheless, it is presumed that the real number is around 500 000, of which half are parasites (Hodda 2022). Certain marine species of this taxon like *Anisakis simplex* and *A. pegreffii* can induce considerable economic losses in the fisheries industry and are important zoonotic agents for public health (Mattiucci et al. 2018; Shamsi and Barton 2023; Cipriani et al. 2024). Fish can act as intermediate, paratenic and definitive hosts for numerous nematode species (Anderson 2000; Pereira and González-Solís 2022). Paratenic hosts occur in the life cycle of numerous helminth parasites (Bush et al. 2001; Parker et al. 2009) and play essential role in increasing the possibility of parasite transmission in favour of its establishment in a site (Marcogliese 2001; Poulin 2007). *Brevimulticaecum* sp. requires arthropods as intermediate hosts (Isaac et al. 2004), fish and other vertebrates as paratenic hosts (Vieira et al. 2010; Ventura et al. 2016) and crocodilians as definitive hosts (Santana et al. 2023).

Anisakid nematodes use several fish species as their paratenic hosts and infect different visceral organs, among which is the liver (Dezfuli et al. 2007; Merhdana et al., 2014; Moravec 2009; Sayyaf Dezfuli et al. 2021a). The effects of nematodes on the liver and condition factors of hosts have been widely reported (Ryberg et al. 2022; Podolska et al. 2024). The intensity of *Anisakis* sp. infection in cod (*Gadus morhua*) livers had a significant negative effect on the condition factors of the host; the infection level increased with the fish length, and higher mortality was observed among large and heavily infected fish (Horbowy et al. 2016). Chronic liver injury leads to liver inflammation and fibrosis, and activated myofibroblasts secrete extracellular matrix proteins that generate fibrous scars (Kisseleva and Brenner 2021). Anisakidae nematode larvae have been found to induce fibrosis in the livers of wild anadromous *Coilia nasus*, upregulating the expression of immunoglobulins IgM and IgD, pro-inflammatory cytokines TNF-α, IL-6 and MCP-1, and associated proteins such as alpha smooth muscle actin, fibronectin and collagen types I and III (Ying et al. 2022). In this study, 2 specimens of *P. flesus* livers exhibited fibrotic scarring. This pathological phenomenon was more frequent in the livers of *G. inaequilabiatus*, most likely because of the higher intensity of infection by *Brevimulticaecum* sp. (L3) (from 4 to 340 worms per organ).

Fish react to extraintestinal helminths by forming connective encapsulation or granulomas (Weber et al. 2022), which are focal chronic inflammatory lesions that appear as nodules in organs harbouring nematode larvae or other helminths (Buchmann and Mehrdana 2016; Molnár et al. 2019; Sayyaf Dezfuli et al. 2021a, 2024; Behrens et al. 2023). Encapsulation is a mutual adaptation between the host immune response and the parasite; it is a strategic compromise that allows the survival of both species (Buchmann 2012). Baltic cod infected with *Contracaecum osculatum* exhibits intense liver inflammation, which manifests as granuloma; this nematode significantly reduces the fat content in hepatocytes and affects the nutritional condition and blood albumin ratio of the body (Santoro et al. 2013; Marnis et al. 2020; Behrens et al. 2023; Podolska et al. 2024). In the same fish–parasite system, the nematode significantly modifies the physiological condition of heavily infected livers (Ryberg et al. 2022). An experimental investigation on the development of another Anisakidae nematode, *Contracaecum rudolphii*, in fishes revealed that the larvae become encapsulated in visceral organs and can survive for at least 18 months to 2 years (Moravec 2009). Information provided in Moravec (2009) on the permanence of nematode larvae in fish visceral organs has been supported by a more recent study (Marnis et al. 2020), showing that Anisakidae nematodes downregulate the synthesis of immune molecules responsible for parasite expulsion. In this study, inflammation, mild fibrosis and fibrotic scarring were observed in the infected livers of flounder and tuvira; these reactions were more severe in the hepatic tissue of tuvira, suggesting that chronic injury had occurred and that most larvae of *Brevimulticaecum* sp. and *A. simplex* (*s.l.*) larvae (L3) had inhabited the livers of these fishes for a long period.

Probably due to the low intensity of infection, the infected flounder livers showed less organ fibrosis, mild hepatocyte rarefaction and a very limited number of lipid droplets, all of which indicate the less severe pathological effects of *A. simplex* (*s.l.*) larvae (L3). Conversely, a higher parasite burden was detected in the tuvira livers, and the hepatocytes showed remarkable rarefaction, with the presence of a high number of lipid droplets in the cytoplasm and abnormal distribution of cell organelles, all symptoms of intense pathological damage induced by *Brevimulticaecum* sp. (L3). Fatty liver is characterized by the aberrant accumulation of lipid droplets, and severe fatty liver in fish can reduce growth performance and feed efficiency, deteriorate meat quality and impair the immune response (Zhu et al. 2025). Anisakidae parasitism activates immune responses and causes liver fibrosis in *C. nasus* fish (Ying et al. 2022). A fibrotic tissue layer with variable thickness was noticed only outside the granuloma around *Brevimulticaecum* sp. (L3), suggesting that this layer might reduce the extent of damage caused by the parasite to the surrounding hepatic tissue of tuvira; such a fibrotic layer was absent around the granulomas which encircled the *A. simplex* (*s.l.*) larvae (L3).

A high number of *C. rudolphii* larvae freely moved and grew mainly in the livers of experimentally infected carp (Moravec 2009). Larger *C. osculatum* larvae migrated in the liver parenchyma of stickleback and goby, where some grew (Køie and Fagerholm 1995); the movement and growth of larvae within the liver are likely feasible due to less or no fibrosis of the organ. Slight fibrosis in the organ and the presence of a translucent space around most *A. simplex* (*s.l.*) larvae (L3) in this study suggest the movement of this nematode in *P. flesus* livers. Conversely, intense fibrosis in the infected livers of tuvira and a lack of translucent space around the larvae of *Brevimulticaecum* sp. (L3) indicate that the parasite was strictly coiled within the granuloma, which impeded worm movement in the organ.

Innate immunity in teleosts relies on various cell types (Secombes and Ellis 2012; Sayyaf Dezfuli et al. 2021b, 2023). Each immune cell type occurring within the granulomas in flounder and tuvira livers infected with *A. simplex* (*s.l.*) larvae (L3) and *Brevimulticaecum* sp. (L3), respectively, is discussed below.

In the flounder–*A. simplex* (*s.l.*) larvae (L3) system, fibro-connective tissue with lesser presence of collagen was observed in the outer layer of the granuloma; mainly fibroblasts were present. Fibroblasts impede the penetration of parasites into host organs (Buchmann 2012), and under pathological conditions, they contribute to the healing of damaged tissues (Secombes and Chappell 1996; Sayyaf Dezfuli et al. 2023; Schuster et al. 2023). An account on fibroblast layers in the granuloma encircling *Brevimulticaecum* sp. (L3) larvae (Sayyaf Dezfuli et al. 2016) and reports on other nematode species (Mólnar et al. 2019; Behrens et al. 2023; Sayyaf Dezfuli et al. 2024) agree with the development of chronic inflammation (Ferguson 2006; Noga 2010). Lymphocytes were observed within the granulomas around the *A. simplex* (*s.l.*) larvae (L3); in some instances, they were in close proximity to the nematode cuticle. The occurrence of lymphocytes in granulomas around nematode larvae in fish has been reported previously (Molnár et al. 2019; Behrens et al. 2023).

Two populations of phagocytes have been identified in fish: neutrophils (Havixbeck et al. 2016) and mononuclear phagocytes (circulating monocytes and tissue macrophages) (Secombes and Ellis 2012; Esteban et al. 2015). Neutrophils are highly motile cells crucial for acute inflammatory responses; they serve as the first line of defence against pathogens (Harvie and Huttenlocher 2015; Havixbeck et al. 2016). The kidney of teleosts has the largest population of neutrophils, which can be rapidly mobilized through blood vessels to sites of inflammation (Havixbeck et al. 2016; Fingerhut et al. 2020). In fish, the chemokine IL-8 acts in recruiting neutrophils (de Oliveira et al. 2013, 2015) and other host immune cells to the site of inflammation (Harvie and Huttenlocher 2015; Jørgensen et al. 2018). The relationship between neutrophils and aquatic pathogens has recently been described (Buchmann 2022). Neutrophils are known to serve as the first line of defence against infiltrating pathogens and are essential to acute inflammatory responses (Havixbeck et al. 2016; Furtado et al. 2019); the presence of neutrophils in the livers of fish infected with nematode larvae has been reported (Dezfuli et al. 2009; Molnár et al. 2019; Ying et al. 2022). In this study, no neutrophils were observed within the granulomas in the flounder and tuvira livers, suggesting that they exhibited chronic injury inflammation and not acute inflammation due to the initiation of infection.

Macrophages play a role in both the innate and adaptive immune system; they are active in the regulation of immune responses and often contain pigments such as lipofuscin, hemosiderin and melanin (Secombes and Ellis 2012; Nathan 2016). They are observed as single macrophages or organized in groups (MAs) or melano-macrophage centres (Agius and Roberts 2003; Stosik et al. 2019). Macrophages exhibit different functional behaviours owing to polarization, which seems to be induced by pathogens or molecules excreted/secreted by them (Lu and Chen 2019). Several functions in fish have been attributed to MAs. The proliferation of MAs is due to both physiological and pathological factors, such as ageing, chemical exposure, starvation and infectious diseases (Couillard et al. 1999). A review of the nature of MAs and their role in fish pathology states that these centres develop focally in association with the late stages of the chronic inflammatory response to severe tissue damage caused by different types of pathogens (Agius and Roberts 2003). Reports on macrophages and MAs in the livers of fish infected with nematode larvae have been published recently (Ying et al. 2022; Behrens et al. 2023). Macrophages and MAs were observed in close proximity to *A. simplex s.l.* larvae (L3) in the parasitized livers of *P. flesus* (Dezfuli et al. 2007); however, the occurrence of such cells was more remarkable in the livers of tuvira harbouring *Brevimulticaecum* sp. (L3) (Sayyaf Dezfuli et al. 2017; present survey). Our previous and current observations support the view that MAs are linked to parasitic infections and represent an inflammatory response (Vogelbein et al. 1987; Dezfuli et al. 2007; Sayyaf Dezfuli et al. 2017).

Fish livers can produce granulomas/capsules formed by metabolically active cells, such as macrophages, and less active cells, such as epithelioid cells (Secombes and Chappell 1996). Epithelioid cells are morphologically similar to epithelial cells; they are transformed macrophages that form upon persistent inflammatory stimulation (Noga et al. 1989; Secombes and Chappell 1996; Gauthier et al. 2004). Necrotic epithelioid cells in close proximity to *A. simplex* (*s.l.*) larvae (L3) and much fewer entities near *Brevimulticaecum* sp. (L3) were observed in flounder and tuvira livers, respectively. The protective wall formed by an inner layer of dead host cells (e.g., epithelioid cells) promotes the survival of larvae (Larsen et al. 2002; Ferguson 2006). The occurrence of necrotic epithelioid cells in the inner layer of granulomas in close proximity to other nematode larvae has been widely reported (Mólnar 1994; Sayyaf Dezfuli et al. 2021a, 2024).

MCs are essential components of host immune systems that perform a secretory function (Reite and Ø 2006; da Silva Wf et al. 2017; Sayyaf Dezfuli et al. 2021b, 2023). Larvae and adult helminths generally induce inflammation in the host digestive tract and associated organs, causing leukocyte migration to the site of infection (Sayyaf Dezfuli et al. 2021b). The close association of MCs with the endothelial cells of capillaries suggests that these cells migrate across the endothelium (Dezfuli and Giari 2008). Acute MC activation is a feature of many types of tissue injury; experimental studies have shown that pathogen/parasite products can activate MCs (Flaño et al. 1996). MCs react to parasites by undergoing degranulation and releasing their contents; this process has been observed in several fish–metazoan systems (Sayyaf Dezfuli et al. 2021b). At the site of infection/inflammation, and in the presence of damaged tissue, MCs release several types of inflammatory mediators, such as piscidins, arachidonic acid metabolites, proteolytic enzymes, cytokines and biogenic amines (Galindo-Villegas et al. 2016; Salger et al. 2016; Douglas et al. 2021; Sayyaf Dezfuli et al. 2021b, 2023). Serotonin and histamine levels in MCs within granulomas formed on the outer surface of eel intestines have been reported (Sayyaf Dezfuli et al. 2024). Serotonin is an important biogenic amine produced and stored in MCs (Serna-Duque and MÁ 2020); it serves as a pro-inflammatory mediator in the infected guts of fish (Sayyaf Dezfuli et al. 2018, 2021b). Histamine was initially detected in the MCs of Perciformes fish (Mulero et al. 2007; Galindo-Villegas et al. 2016) and was subsequently found in the enteric immune cells of other fish species that harboured parasites (Sayyaf Dezfuli et al. 2018). In this study, very few MCs were observed in granulomas in infected *P. flesus* livers; in contrast, parasitized *G. inaequilabiatus* livers exhibited more numerous MCs that were arranged in some layers, mainly in the middle layer of the granulomas.

The results of the current study show that (a) larvae of different nematode species in distinct host species do not elicit the same histopathological damage; (b) *A. simplex* (*s.l.*) larvae (L3) did not induce very intense pathological alterations in flounder livers and (c) *Brevimulticaecum* sp. (L3) provoked severe damage in tuvira livers, and focal encapsulation of the nematode allowed uninfected portions of the organs to maintain their functions and permit the survival of the host, and thereby, the parasite.
